# Structure‐Function Integration in 2D Hybrid Perovskite for Fast Neutron and Gamma Ray Discrimination

**DOI:** 10.1002/advs.202518905

**Published:** 2025-12-14

**Authors:** Yingming Wang, Lingyan Xu, Chongqi Liu, Lu Liang, Binghui Zhang, Zhentao Qin, Lixiang Lian, Wei Zheng, Yanyan Lei, Qinzeng Hu, Shuai Song, Chaopeng Mi, Tao Wang, Yadong Xu, Gangqiang Zha, Wanqi Jie

**Affiliations:** ^1^ State Key Laboratory of Solidification Processing School of Materials Science and Engineering Northwestern Polytechnical University Xi'an 710072 China; ^2^ MIIT Key Laboratory of Radiation Detection Materials and Devices School of Materials Science and Engineering Northwestern Polytechnical University Xi'an 710072 China; ^3^ Science and Technology on Thermostructural Composite Materials Laboratory School of Materials Science and Engineering Northwestern Polytechnical University Xi'an 710072 China; ^4^ Research and Development Institute of Northwestern Polytechnical University in Shenzhen Shenzhen 518057 China

**Keywords:** (GABA)_2_PbBr_4_ single crystals, fast neutron detection, hybrid lead halide perovskites, pulse shape discrimination, scintillation imaging

## Abstract

Efficient discrimination between fast neutrons and gamma rays is crucial yet challenging for radiation detection. Here, the successful growth of large‐size, high‐quality 2D organic–inorganic hybrid perovskite single crystals, (GABA)_2_PbBr_4_, via an in situ seed‐assisted cooling method is reported. This material integrates hydrogen‐rich GABA cations with heavy‐atom lead halide layers, offering strong excitonic emission, efficient γ‐ray absorption, and fast neutron sensitivity. X‐ray diffraction reveals highly oriented crystals along the (004) plane with a narrow full width at half maximum (FWHM) of 12.81″. Optical characterizations indicate a wide bandgap (≈3.0 eV), sharp excitonic emission, and prominent self‐trapped exciton behavior, supported by transient and temperature‐dependent photoluminescence. The crystals exhibit an energy resolution of 9.0% at 662 keV and a high light yield of 10 695 ph MeV^−1^. Notably, superior neutron/gamma pulse shape discrimination (PSD) is achieved with a maximum figure‐of‐merit (FOM) of 1.74, enabling clear signal separation. Additionally, flexible composite scintillation films based on (GABA)_2_PbBr_4_ demonstrate high‐resolution X‐ray imaging up to 10 LP mm^−1^. These results highlight the potential of (GABA)_2_PbBr_4_ as a multifunctional scintillator for advanced radiation detection and imaging applications.

## Graphical abstract

Using an in situ seed‐assisted cooling method, large (28 × 7 × 3 mm^3^) (GABA)_2_PbBr_4_ single crystals are grown, featuring hydrogen‐rich organic layers and a dense PbBr_4_ framework. This architecture enables efficient γ‐ray absorption, fast‐neutron sensitivity, and clear n/γ pulse‐shape discrimination (FOM up to 1.74), highlighting its potential as a multifunctional scintillator.

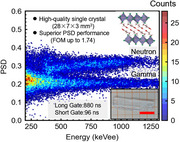

## 1 Introduction

Neutron detection plays an irreplaceable role in a wide range of critical fields, including nuclear security, radiation monitoring, reactor diagnostics, and particle physics.^[^ ^1^, ^2^ ^]^ However, due to their lack of electric charge, neutrons cannot directly induce excitation or ionization via electromagnetic interactions and must instead be detected through secondary reactions with atomic nuclei.^[^ ^3^, ^4^, ^5^ ^]^ This challenge is further compounded by the fact that most neutron detection environments are accompanied by intense γ‐ray backgrounds, as these photons can also excite scintillators to produce signals that are often difficult to distinguish from neutron events.^[^ ^6^ ^]^ Consequently, developing scintillation materials that offer both high sensitivity to neutrons and excellent n/γ discrimination remains a central goal in the advancement of neutron detection technologies.

Different detection strategies have been established for neutrons across distinct energy regimes. Thermal neutrons (E < 0.5 eV) are typically detected through capture reactions involving isotopes such as ^10^B, ^6^Li, or ^3^He, which release charged particles (e.g., α particles, tritons) that are readily measurable.^[^ ^7^, ^8^, ^9^ ^]^ In contrast, fast neutrons (E > 100 keV) are more challenging to detect due to their low capture cross‐sections and are generally detected via elastic scattering with light nuclei. In this process, a neutron transfers kinetic energy to a target nucleus, which recoils with an energy given by:^[^ ^10^ ^]^

(1) $$\frac{E_{R}}{E_{N}}=\frac{4A}{{\left(A+1\right)}^{2}}cos^{2}\theta$$

where *E_N_* is the incident neutron energy, *E_R_* is the recoil nucleus energy, *A* is the atomic mass of the target nucleus, and θ is the recoil angle. As Equation (1) shows, hydrogen nuclei enable the highest energy transfer, making hydrogen‐rich media essential for fast neutron detection. Owing to their high hydrogen content, fast response times, and effective pulse shape discrimination (PSD) capabilities, organic scintillators have become the primary choice for fast neutron detection. Prototypical materials such as stilbene and EJ‐301 have long served as standards in this field.^[^ ^11^, ^12^, ^13^, ^14^, ^15^ ^]^ However, these materials exhibit nonlinear energy responses to fast neutrons and low‐energy charged particles, complicating neutron spectroscopy. Moreover, their relatively low densities and atomic numbers limit their γ‐ray absorption efficiency, reducing spectral resolution in complex radiation environments.

Inorganic scintillators such as NaI and CsI, by contrast, possess high density and atomic number, making them excellent absorbers of γ‐rays.^[^ ^16^, ^17^ ^]^ However, the absence of hydrogen atoms renders them largely ineffective for fast neutron detection. This intrinsic contradiction between the need for low‐Z, hydrogen‐rich elements for neutron detection and high‐Z elements for γ‐ray absorption presents a fundamental challenge in realizing dual‐mode n/γ detection scintillators. Addressing this dilemma requires the development of multifunctional materials that simultaneously combine high neutron sensitivity, strong γ‐ray attenuation, and excellent PSD performance.

In this context, organic–inorganic hybrid lead halide perovskites have emerged as a promising class of materials for radiation detection due to their tunable compositions and structures, outstanding optical properties, and favorable radiation responses. On one hand, the incorporation of organic chains introduces abundant hydrogen atoms that enhance fast neutron sensitivity through elastic scattering. On the other hand, the heavy elements in the lead‐halide framework provide effective γ‐ray absorption. Recent studies have demonstrated the potential of these 2D perovskites in fast neutron detection. For example, Wan et al. reported that (BA)_2_PbBr_4_ and (PEA)_2_PbBr_4_ exhibit linear responses to fast neutrons, with photoluminescence dominated by the lead‐halide octahedral framework, whose high density of states helps suppress non‐radiative quenching.^[^ ^18^ ^]^ Xiang et al. achieved n/γ discrimination in undoped (PEA)_2_PbBr_4_ single crystals, with a figure‐of‐merit (FOM) of 0.73, and further improved it to 1.03 by Sb^3+^ doping.^[^ ^19^ ^]^ Although these results are encouraging, the FOM still falls short of the conventional benchmark of 1.27 for effective n/γ separation in organic scintillators,^[^ ^20^ ^]^ indicating that there remains significant room for improvement.

The suboptimal FOM values observed in current 2D perovskite scintillators are determined not only by their intrinsic emission properties but also by their limited particle absorption capabilities. Since the detection of fast neutrons and γ‐rays fundamentally relies on sufficient interaction with the incident radiation, the growth of large and thick single crystals is essential for practical detector applications.^[^ ^21^ ^]^ However, due to the inherently weak van der Waals interactions between adjacent organic layers, 2D perovskites tend to grow laterally, resulting in thin, plate‐like crystals with restricted thickness.^[^ ^22^ ^]^ This morphology reduces the effective detection volume and significantly weakens the absorption of high‐energy photons and neutrons, thereby limiting their performance in fast neutron and γ‐ray detection systems.^[^ ^23^ ^]^ Furthermore, the fragile interlayer bonding undermines mechanical robustness and long‐term structural stability, posing additional challenges for scalable device fabrication and stable detector operation.^[^ ^24^ ^]^ Overcoming these morphological and structural limitations is thus critical for fully unlocking the potential of 2D perovskites in high‐performance radiation detection applications.

To address these limitations, organic cations bearing functional groups such as hydroxyl or carboxyl moieties have been introduced to promote hydrogen bonding between inorganic layers and enhance interlayer cohesion.^[^ ^25^, ^26^ ^]^ In systems such as (EA)_2_PbI_4_ (EA = HOC_2_H_4_NH_3_), the presence of hydroxyl groups facilitates the formation of interlayer hydrogen‐bonding networks, which not only enhance environmental stability but also significantly affect the exciton binding energy.^[^ ^26^, ^27^ ^]^ The formation of carboxylic acid dimers across neighboring inorganic slabs introduces strong interlayer hydrogen bonds, which have been associated with structural distortion and broadband white‐light emission in some 2D perovskites.^[^ ^28^, ^29^ ^]^ A representative example is (AVA)_2_PbCl_4_, based on 5‐ammonium valeric acid (AVA), where the presence of carboxyl‐mediated hydrogen bonding induces notable octahedral distortion, resulting in efficient white‐light emission.^[^ ^28^ ^]^ Similarly, in (t‐ACH)_2_PbI_4_ (t‐ACH = HOOC–(CH_2_)_8_–NH_3_), the hydrogen‐bonded dimerization of carboxylic acids significantly enhances both environmental and thermal stability.^[^ ^30^ ^]^ γ‐Aminobutyric acid (GABA), a linear amino acid featuring both amino and carboxyl groups, has emerged as a promising organic cation. Compared with conventional bulky cations such as PEA, GABA can form denser and stronger interlayer hydrogen bonds, thereby enhancing the overall structural robustness and crystal quality.^[^ ^25^, ^31^ ^]^ In particular, single crystals of (GABA)_2_PbBr_4_ have achieved a high photoluminescence quantum yield (PLQY) of up to 21.2%,^[^ ^31^ ^]^ highlighting their potential in scintillation applications. However, the same strong hydrogen‐bonding affinity of GABA that benefits structural cohesion also presents challenges during crystal growth. In hydrobromic acid solution, GABA tends to form stable hydrogen bonds with water molecules, increasing the nucleation barrier.^[^ ^32^ ^]^ As a result, crystal nucleation only occurs under high supersaturation conditions, often leading to rapid and excessive nucleation. This behavior makes it difficult to grow large, high‐quality single crystals.

In this work, an in situ seed‐assisted cooling‐growth method is developed to synthesize large (GABA)_2_PbBr_4_ single crystals with centimeter‐scale dimensions and high crystallinity. The crystallinity and photoluminescence properties of (GABA)_2_PbBr_4_ crystals were comprehensively characterized, and their light output was quantitatively evaluated using relative pulse height analysis. Additionally, the γ‐ray detection performance and n/γ PSD capability of (GABA)_2_PbBr_4_ detectors were systematically investigated at room temperature. Finally, flexible composite films embedding (GABA)_2_PbBr_4_ in PDMS were fabricated to explore their X‐ray imaging potential. These results establish (GABA)_2_PbBr_4_ as a promising multifunctional scintillator for both fast neutron and X‐ray detection, offering a new design paradigm for next‐generation radiation sensing and imaging platforms.

## 2 Results and Discussion

### 2.1 In Situ Seed‐Assisted Crystal Growth and Structural Characterization

**Figure 1:**
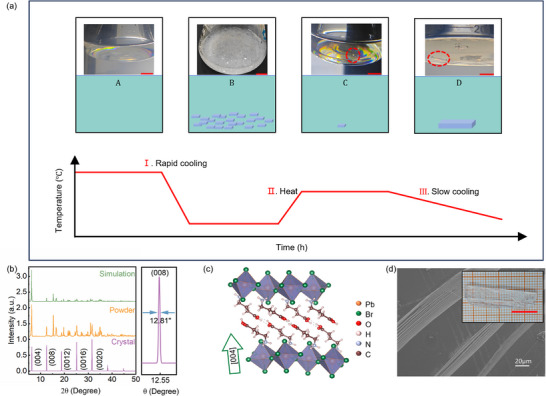
In situ seed‐assisted crystallization strategy and structural characterization of (GABA)_2_PbBr_4_ single crystals. a) Schematic illustration of the in situ seed‐assisted cooling crystallization process. Scale bar: 10 mm (red). b) Experimental and simulated powder XRD patterns of (GABA)_2_PbBr_4_, along with the high‐resolution rocking curve of the (008) plane. c) Schematic diagram of the layered stacking structure of (GABA)_2_PbBr_4_. d) SEM image showing the layered texture along the (004) direction. Inset: photograph of a centimeter‐scale single crystal with high optical quality. Scale bar: 10 mm (red).

Precise control over nucleation and growth is critical for the solution‐based synthesis of high‐quality single crystals. To address the challenges above, an in situ seed‐assisted cooling crystallization strategy was developed. By regulating temperature variation and solution supersaturation, in situ seeds were introduced to guide the growth process. This approach enabled the synthesis of large (GABA)_2_PbBr_4_ single crystals with high crystallinity. The process consists of four key steps (**Figure** **1**a): first, GABA and PbBr_2_ were dissolved in hydrobromic acid at 60 °C in a stoichiometric ratio to form a clear and homogeneous precursor solution. Second, rapid cooling was applied to induce the initial precipitation of numerous small crystals. Third, slow reheating partially dissolved these crystals, leaving behind a few stable microcrystals to serve as seeds. Finally, a gradual cooling step facilitated sustained growth from a single seed crystal, ultimately yielding centimeter‐scale single crystals.

As shown in Figure 1b, the powder X‐ray diffraction (XRD) pattern of the product closely matches the simulated spectrum, confirming the formation of the desired phase. High‐resolution XRD measurements reveal sharp diffraction peaks with a narrow full width at half maximum (FWHM) of only 12.81″, indicating excellent crystallinity. Single‐crystal XRD patterns exhibit sharp diffraction along the (004) planes, indicating a preferential layered orientation (Figure 1c), which is further confirmed by optical microscopy (Figure S1, Supporting Information) and scanning electron microscopy (Figure 1d). The inset of Figure 1d shows a photograph of the as‐grown crystal, with dimensions of 28 × 7 × 3 mm^3^, highlighting its centimeter‐scale size and high optical quality.

To better understand the challenges in controlling nucleation during the growth process, nucleation kinetics were analyzed using classical theory. Crystallization is driven by supersaturation, which reflects the chemical potential difference between solute molecules in the solution and in the crystal phase. As temperature increases, the nucleation driving force rises, leading to precipitation under supersaturated conditions. This driving force is proportional to ln (*S*).^[^ ^33^ ^]^ The nucleation rate *J* follows an Arrhenius‐type behavior:^[^ ^34^ ^]^

(2) $$J=\Lambda \exp\left(\frac{-\Delta G}{k_{B}T}\right)$$

where Λ depends on supersaturation, *k_B_* is the Boltzmann constant and *T* is the temperature. The nucleation barrier Δ*G* can be expressed as:^[^ ^32^ ^]^

(3) $$\Delta G=\frac{16}{3}\pi \sigma^{3}{\left(\xi -\xi_{A}-E_{C}+k_{B}T\ln\frac{1}{\frac{{M_{S}}^{j}}{M_{A}}-j^{2}{M_{S}}^{j-1}}\right)}^{-2}$$

where σ is the surface free energy, ξ and ξ_*A*_ represent the cohesive energy of precursor molecules in the crystalline lattice and in the free (dissolved) state, respectively, *E_C_* is the binding energy between solute and solvent molecules, and *M_S_*, *M_A_* are the molar concentrations of solvent and precursor. The exponent *j* accounts for the number of solvent molecules interacting with each precursor species.

According to Equation (3), the nucleation barrier Δ*G* is governed by the synergistic interplay of multiple thermodynamic parameters. When (GABA)_2_PbBr_4_ precursors are dissolved in aqueous HBr, the polar –NH_3_^+^ and –COO^−^ groups of GABA^+^ form strong hydrogen bonds with water molecules. This interaction directly enhances the solute–solvent binding energy, significantly increasing *E_C_*. Thermodynamically, this elevated *E_C_* reduces the net driving force, thereby raising the nucleation barrier. Simultaneously, the strong hydrogen bonding promotes the formation of a dense hydration shell around the solute (implying a large solvation number, *j*). This enhanced solvation further stabilizes the dissolved state and decreases the concentration of free, unbound solute species (*M_A_*). The resulting reduction in effective chemical potential difference between the solvated and crystalline states suppresses nucleation even further. Consequently, overcoming this compounded energetic barrier requires the system to reach a considerably higher level of supersaturation before nucleation can occur. However, this can trigger sudden burst nucleation, which impedes the controlled growth of large, high‐quality single crystals. Although introducing seed crystals is a common strategy to regulate growth, the strong hydrogen bonding also enhances the solubility of seeds in the solvent. As a result, even minor fluctuations during sealing or thermal holding can lead to seed dissolution, severely affecting subsequent crystal growth. The in situ seed‐assisted strategy effectively addresses these challenges by stabilizing seed crystals and controlling nucleation dynamics, enabling the reproducible synthesis of centimeter‐scale (GABA)_2_PbBr_4_ single crystals with excellent crystallinity.

### 2.2 Photophysical Properties and Exciton Dynamics

**Figure 2:**
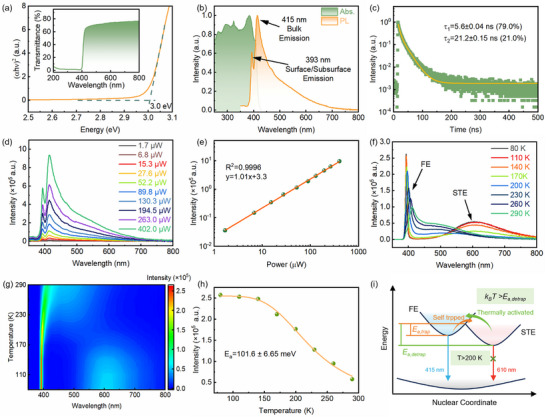
Optical properties and exciton dynamics of (GABA)_2_PbBr_4_ single crystals. a) UV–vis transmission spectrum (inset) and Tauc plot of (GABA)_2_PbBr_4_ single crystals. b) Comparison of UV–vis absorption and photoluminescence (PL) spectra under 325 nm excitation. c) Transient photoluminescence decay curve of the 415 nm emission fitted with a bi‐exponential function. d) Power‐dependent PL spectra. e) Integrated PL intensity as a function of excitation power. f) Temperature‐dependent PL spectra. g) Corresponding contour plot of the temperature‐dependent PL spectra. h) Temperature‐dependent PL intensity at 415 nm fitted using an exciton binding energy model. i) Schematic illustration of exciton dynamics and radiative pathways including FE and STE.

The ultraviolet–visible (UV–vis) transmission spectrum of (GABA)_2_PbBr_4_ single crystals is shown in the inset of **Figure** **2**a. A sharp absorption edge appears at ≈400 nm, and the optical transmittance exceeds 70% in the 500–800 nm range, indicating excellent crystallinity and low defect density. The optical bandgap, extracted from the Tauc plot in Figure 2a, is estimated to be ≈3.0 eV. Figure 2b compares the UV–vis absorption spectrum with the photoluminescence (PL) spectrum under 325 nm excitation. The PL spectrum displays two sharp emission peaks at 393 and 415 nm (the latter being dominant), accompanied by a broad low‐energy emission. This broad emission is attributed to radiative recombination from self‐trapped excitons (STEs) and lattice defects. Structural analysis (Figure S2, Supporting Information) reveals a significantly distorted octahedral framework, with a Br–Pb–Br bond angle of 146.0°, far from the ideal 180°, suggesting enhanced electron–phonon coupling that facilitates STE formation.

In addition, the dual emission peaks at 393 and 415 nm in the high‐energy region may originate from bandgap variations between the surface and bulk regions of the crystal, resulting in dual‐state emission.^[^ ^35^, ^36^ ^]^ Due to the limited penetration depth of 325 nm excitation, the luminescence primarily probes the near‐surface region, where surface states are more likely to contribute to the emission. In contrast, under X‐ray excitation—characterized by a significantly larger excitation volume—only the 415 nm peak is observed (Figure S3, Supporting Information), indicating that this emission predominantly arises from the crystal bulk. Furthermore, the spectral overlap between the PL and absorption spectrum suggests potential self‐absorption effects, which could reduce the external photoluminescence efficiency.^[^ ^37^ ^]^ Transient PL measurements for the 415 nm emission under 400 nm excitation are presented in Figure 2c. The decay curve is well fitted with a bi‐exponential function:^[^ ^38^ ^]^

(4) $$I\left(t\right)=A+\sum_{i}B_{i}e^{\left(-\frac{t}{\tau_{i}}\right)}$$

resulting in lifetimes of τ_*PL*, *Fast*_ = 5.6 ± 0.04 ns(79.0%) and τ_*PL*,*Slow*_ = 21.2 ± 0.15 ns(21.0%). The faster decay component is attributed to nonradiative recombination associated with surface defects, whereas the slower component corresponds to radiative recombination within the crystal bulk.^[^ ^36^ ^]^

Power‐dependent PL spectra (Figure 2d) indicate that the 415 nm emission intensity increases with excitation power. The integrated emission intensity follows an approximately linear power‐law dependence (*I*∝*P^n^*, with n ≈ 1, R^2^ = 0.9996) as shown in Figure 2e, suggesting that the emission is primarily governed by free excitons (FE) or STE.^[^ ^39^ ^]^

Figure 2f,g presents the temperature‐dependent PL spectra and corresponding contour plots. With increasing temperature, the main emission peak exhibits a redshift accompanied by a significant intensity decrease, indicating pronounced thermal quenching of exciton recombination. The temperature‐dependent PL intensity at 415 nm was fitted using an exciton binding energy model:^[^ ^40^ ^]^

(5) $$I\left(T\right)=\frac{I_{0}}{1+Aexp\left(-\frac{E_{a}}{kT}\right)}$$

as shown in Figure 2h, yielding an exciton binding energy *E_a_* = 101.6 ± 6.65 meV. This confirms the stable existence of excitons at room temperature in (GABA)_2_PbBr_4_ single crystals, consistent with their good optical stability and radiative efficiency.^[^ ^41^, ^42^ ^]^

At 80 K, an additional broad emission band centered ≈610 nm appears in the PL spectrum, which is only observed at low temperatures, indicating its origin from STE radiative recombination.^[^ ^40^, ^43^ ^]^ Upon optical excitation, electron–hole pairs first form FE. A fraction of these excitons become self‐trapped in lattice‐distorted regions due to strong electron–phonon coupling, resulting in STE states.^[^ ^44^ ^]^ At room temperature, the thermal energy of the lattice is sufficient to thermally activate the de‐trapping of STE excitons back to the FE state, leading to fast radiative recombination and dominant narrowband emission at 415 nm, thereby suppressing the broad STE emission. As the temperature decreases below 200 K, thermal activation weakens, hindering the de‐trapping of STE excitons and increasing their radiative recombination probability, which enhances the broad emission at 610 nm. The related exciton dynamics and radiative pathways are illustrated in Figure 2i.

### 2.3 Neutron and Gamma Ray Pulse Shape Discrimination

**Figure 3:**
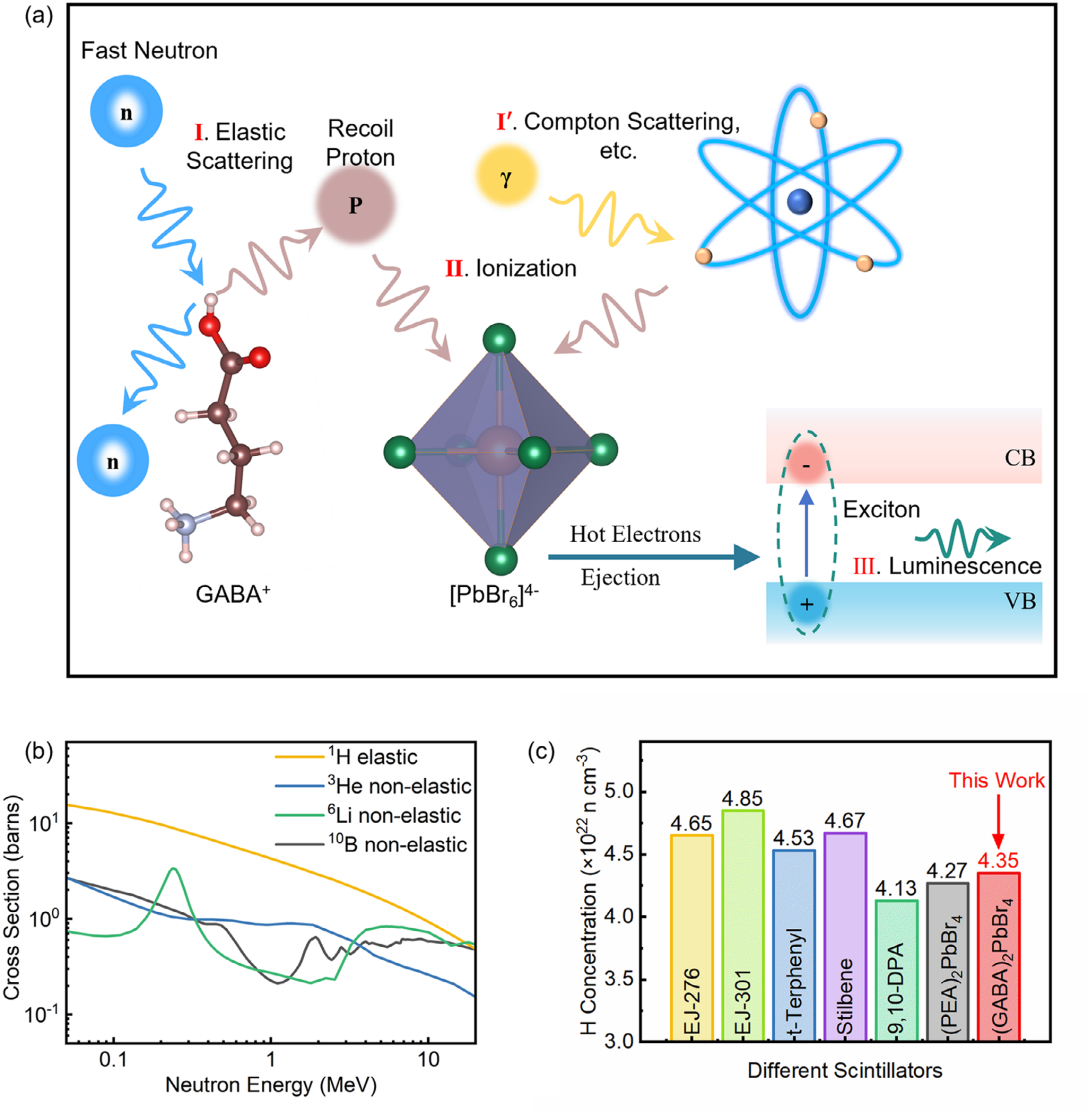
Neutron and gamma ray PSD of (GABA)_2_PbBr_4_ single crystals. a) Schematic of fast neutron and gamma ray interaction mechanisms, showing stages of elastic scattering, ionization, exciton formation, and luminescence. b) Neutron interaction cross sections of ^1^H, ^3^He, ^6^Li, and ^10^B. c) Hydrogen content comparison among (GABA)_2_PbBr_4_, typical organic scintillators, and 2D perovskite (PEA)_2_PbBr_4_.

**Figure** **3**a illustrates the schematic interaction mechanisms of fast neutrons and gamma rays in (GABA)_2_PbBr_4_ single crystals. The fast neutron detection process can be divided into three typical stages: (I) elastic scattering; (II) ionization; and (III) excitation and luminescence. In stage (I), incident neutrons undergo elastic scattering with hydrogen atoms in the organic chains, transferring part of their kinetic energy to recoil protons. In stage (II), these recoil protons induce strong ionization in the [PbBr_6_]^4−^ octahedral framework, generating a large number of hot electrons. These electrons rapidly thermalize near the conduction band minimum (CB) and valence band maximum (VB), forming electron–hole pairs. Driven by Coulomb interaction, these carriers recombine to form excitons. In stage (III), excitons undergo radiative recombination, emitting scintillation photons that constitute detectable optical signals. In contrast, the γ‐ray detection mechanism begins with interactions between γ photons and the [PbBr_6_]^4−^ octahedra, including photoelectric effect, Compton scattering, and electron‐positron pair production (stage I′), releasing numerous secondary electrons. These secondary electrons then undergo excitation, thermalization, and exciton formation, ultimately producing detectable luminescence. Due to differences in the energy deposition paths, density, and temporal evolution characteristics between recoil protons and secondary electrons, the fluorescence signals they induce exhibit distinguishable temporal features. This provides the physical basis for effective PSD between fast neutron and γ‐ray events.^[^ ^38^ ^]^

Figure 3b compares the fast neutron interaction cross sections of key nuclides, including ^1^H, ^3^He, ^6^Li, and ^10^B, highlighting the dominant role of hydrogen via its large elastic scattering cross section. Among these candidates, hydrogen is particularly effective for facilitating elastic energy transfer and enabling PSD‐capable scintillation. Figure 3c shows the hydrogen atom concentrations in (GABA)_2_PbBr_4_ compared with typical organic scintillators (such as EJ‐301) and the 2D hybrid perovskite (PEA)_2_PbBr_4_. The results show that the hydrogen content in (GABA)_2_PbBr_4_ is comparable to that of traditional organic scintillators and even slightly higher than that in (PEA)_2_PbBr_4_, indicating that it possesses a good hydrogen content foundation for fast neutron detection and is promising for achieving excellent n/γ discrimination performance.

**Figure 4:**
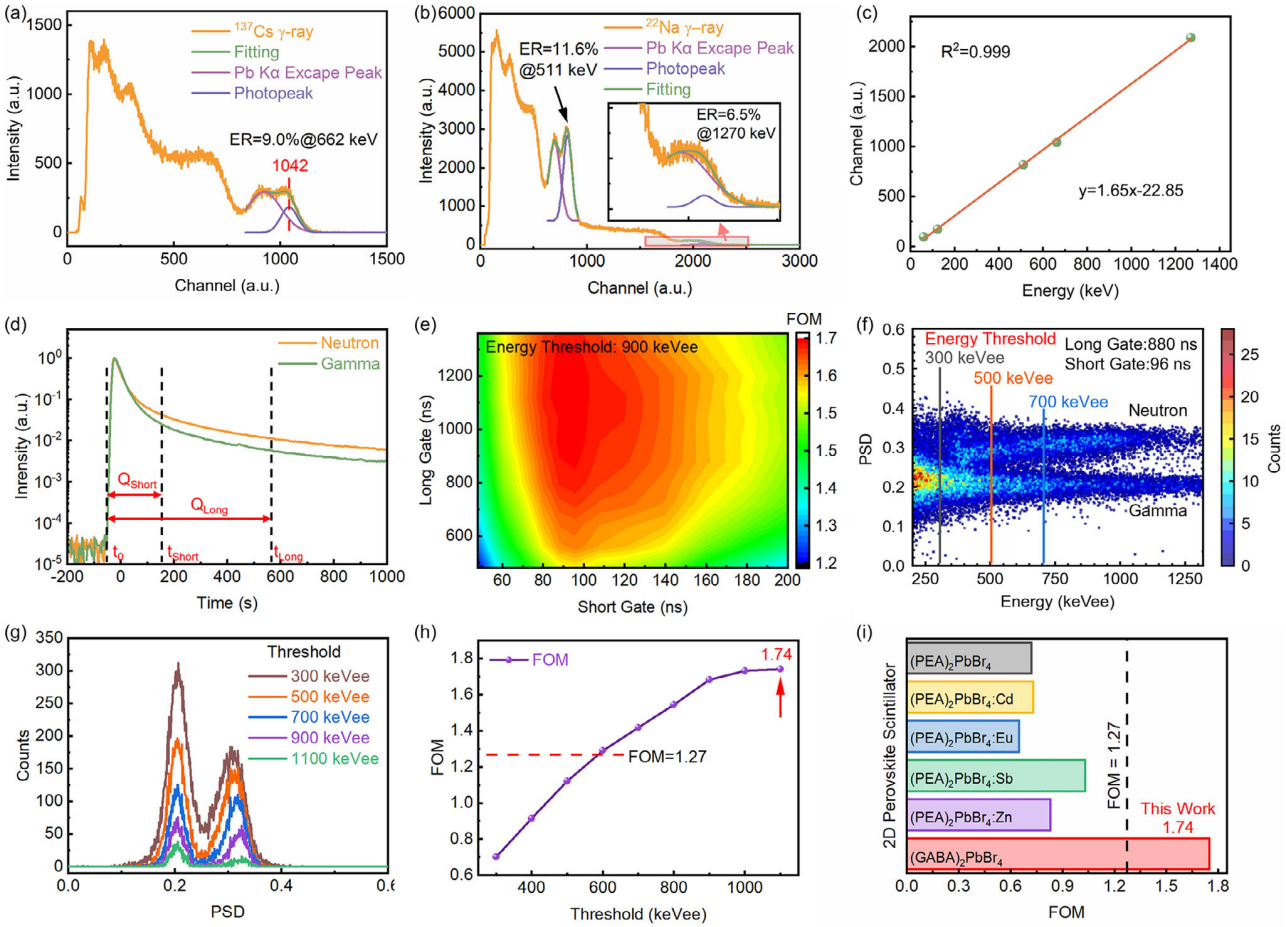
Gamma response and n/γ PSD performance of (GABA)_2_PbBr_4_ single crystal. a) Pulse height spectrum under 662 keV γ‐rays from a ^137^Cs source. b) Pulse height spectra under 511 and 1274 keV γ‐rays from a ^22^Na source. c) Gamma response linearity over a wide energy range. d) Typical pulse waveforms under fast neutron and γ‐ray excitation. e) Optimization of short and long gate widths for FOM. f) PSD scatter plot at optimized gate settings. g) PSD distributions at different energy thresholds. h) FOM versus energy threshold showing n/γ discrimination. i) Comparison of PSD performance between (GABA)_2_PbBr_4_ and 2D hybrid perovskite scintillators.^[^ ^19^, ^37^, ^38^ ^]^

**Figure** **4**a presents the pulse height spectrum of a (GABA)_2_PbBr_4_ single‐crystal scintillator under 662 keV γ‐ray irradiation from a ^137^Cs source, showing an energy resolution of 9.0%. The photopeak and Pb escape peaks are clearly resolved, reflecting the excellent crystal quality resulting from the in situ seed‐assisted growth strategy, which ensures uniform crystallization and defect control. The absolute light yield was evaluated using a relative pulse height method, with NaI:Tl scintillators as a reference (Figure S4, Supporting Information), yielding a value of 10 695 ph MeV^−1^ for (GABA)_2_PbBr_4_. Further performance tests with a ^22^Na source emitting two γ‐rays (511 and 1274 keV) are shown in Figure 4b, where the scintillator accurately resolves both photo‐peaks with energy resolutions of 11.6% and 6.5%, respectively. To systematically evaluate γ‐ray response linearity, the scintillator was tested with standard γ sources at various energies (^241^Am, ^57^Co, ^137^Cs, and ^22^Na), as displayed in Figures 4a–c and S5 (Supporting Information). The results demonstrate a good linear response of the (GABA)_2_PbBr_4_ single crystal across a wide energy range.

Figure 4d shows typical pulse waveforms recorded under fast neutron and γ‐ray excitation. To analyze the luminescence kinetics under different excitations, the pulse decay was fitted using a bi‐exponential function (Equation 4), extracting the fast and slow component time constants (τ) and their relative contributions (R), summarized in **Table** **1**.

**Table 1:** Statistical information on GPB response times to gamma rays and neutrons.

Type	τ_*Fast*_ (ns)	*R_Fast_* (%)	τ_*Slow*_ (ns)	*R_Slow_* (%)
Neutron	21.1 ± 0.12	49	140.9 ± 2.38	51
Gamma	22.4 ± 0.13	67	126.2 ± 3.56	33

Under neutron excitation, the fast component has a decay constant of 21.1 ± 0.12 ns, accounting for 49% of the signal, while the slow component exhibits 140.9 ± 2.38 ns with 51% contribution. In contrast, γ‐ray excitation yields a slightly longer fast decay (22.4 ± 0.13 ns) with a higher fraction (67%) and a slow component of 126.2 ± 3.56 ns accounting for 33%. These differences indicate distinct luminescence kinetics for neutron‐ and γ‐ray‐induced excitations in (GABA)_2_PbBr_4_, originating from their fundamentally different energy deposition mechanisms. Neutrons produce highly localized exciton populations via elastic scattering with hydrogen atoms, leading to enhanced bi‐exciton or multi‐exciton interactions that accelerate nonradiative recombination, resulting in a faster fast decay component.^[^ ^45^ ^]^

Simultaneously, the high exciton density promotes exciton‐defect interactions, increasing trapping/de‐trapping processes and thus enhancing the slow decay component.^[^ ^46^, ^47^ ^]^ Moreover, these decay behaviors differ from TRPL results under 400 nm laser excitation, where limited penetration depth leads to surface‐dominated signals characterized by τ_*PL*, *Fast*_ = 5.6 ± 0.04 ns and τ_*PL*,*Slow*_ = 21.2 ± 0.15 ns, reflecting low‐excitation conditions near the surface. In contrast, neutron and γ excitations generate higher exciton densities and deeper excitation profiles, thus probing bulk recombination dynamics.

To achieve effective n/γ discrimination, the Charge Comparison Method (CCM) was employed for PSD measurements.^[^ ^48^ ^]^ This method calculates the charge integrals over two different time gates: short gate (*Q_Short_*) and long gate (*Q_Long_*), defining PSD as:

(6) $$\mathrm{PSD}=\frac{Q_{Long}-Q_{Short}}{Q_{Long}}$$

As shown in Figure 4d, the gate settings critically affect discrimination performance; thus, optimizing the integration windows is essential for the overall detector efficacy. The FOM is commonly used to evaluate the separation degree between neutron and gamma ray distributions, defined as:^[^ ^48^ ^]^

(7) $$FOM=\frac{\left|P_{n}-P_{\gamma }\right|}{FWHM_{n}+FWHM_{\gamma }}$$

where *P_n_* and *P*_γ_ denote the centroids of Gaussian fits to the neutron and gamma ray PSD peaks, respectively, and *FWHM_n_* and *FWHM*_γ_ are the full widths at half maximum.

In the equation, *P_n_* and *P*_γ_ represent the centroid positions of the Gaussian‐fitted neutron and γ‐ray peaks in the PSD histogram, while *FWHM_n_* and *FWHM*_γ_ correspond to the full widths at half maximum of the neutron and γ‐ray peaks, respectively. Larger FOM values indicate better n/γ discrimination, with values above 1.27 representing complete separation.^[^ ^20^ ^]^

To optimize gate settings, the effect of varying long gates (480–1360 ns) and short gates (48–200 ns) was studied at a fixed energy threshold of 900 keVee, as shown in Figure 4e. Results indicate the FOM initially increases and then decreases with short gate duration, peaking at 96 ns, while longer integration windows steadily improve FOM until saturating ≈880 ns. Hence, the optimal gates were chosen as 96 ns (short) and 880 ns (long). The PSD distribution under these conditions (Figure 4f) clearly separates neutron and gamma ray events. Additionally, PSD distributions at different energy thresholds (Figure 4g) show increasing n/γ separation with higher energy. Figure 4h plots the FOM versus energy threshold, with discrimination significantly improving above 600 keVee and reaching a maximum FOM of 1.74 at 1100 keVee, demonstrating complete n/γ separation. To comprehensively evaluate the practical potential of (GABA)_2_PbBr_4_, we compared its key scintillation parameters with representative organic and inorganic scintillators, as well as other 2D hybrid perovskites, as detailed in Figure 4i and Table S1 (Supporting Information). As shown in the comparison, the FOM of 1.74 achieved in this work significantly outperforms other 2D perovskite counterparts, highlighting the structural advantage conferred by the hydrogen‐rich GABA cations. Although its light yield is lower than that of some commercial inorganic scintillators, it is comparable to that of organic scintillators. Crucially, (GABA)_2_PbBr_4_ demonstrates a distinct advantage in dual‐mode fast neutron and γ‐ray detection compared to purely organic or inorganic options. By combining efficient pulse shape discrimination with resolved γ‐ray spectroscopy, it effectively bridges the functional gap between organic and inorganic scintillators.

### 2.4 X‐ray Imaging Performance of (GABA)_2_PbBr_4_@PDMS Flexible Film

**Figure 5:**
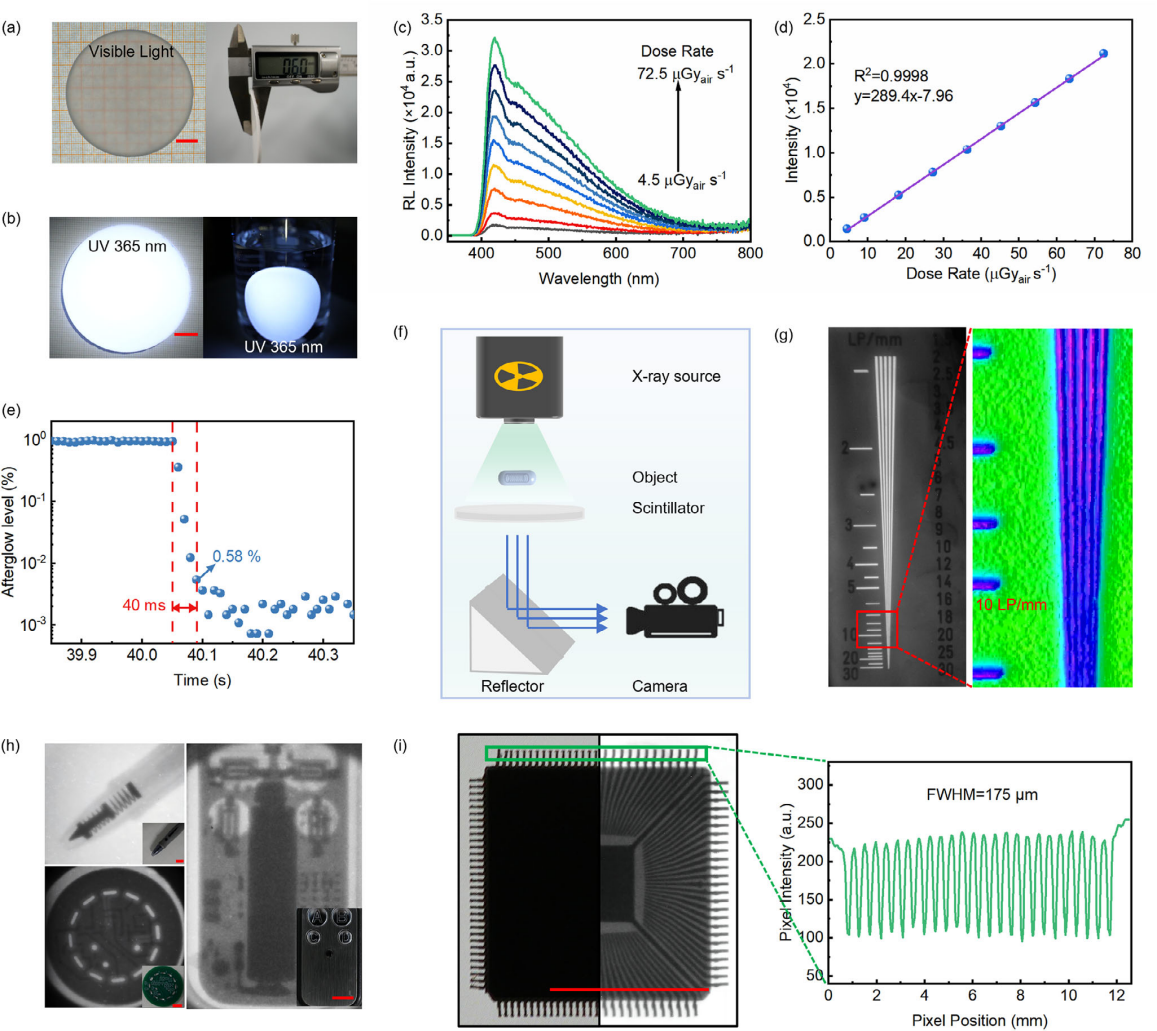
X‐ray imaging performance of the (GABA)_2_PbBr_4_@PDMS flexible film. a) Photograph of the flexible composite film under visible light showing uniformity and optical transparency. b) Bright white fluorescence emission of the film under UV excitation and in water. c) Radioluminescence (RL) intensity of the film under different X‐ray dose rates. d) Linear correlation between RL intensity and X‐ray dose rate. e) Rapid decay of the RL signal after termination of X‐ray exposure. f) Schematic diagram of the X‐ray imaging system using the flexible film. g) X‐ray image of a standard line pair phantom showing >10 LP mm^−1^ resolution. h) X‐ray images of various objects, including a pen, a chip, and a remote control. Scale bar: 10 mm (red). i) X‐ray image of the chip. The spatial resolution was determined by fitting the grayscale intensity profile (along the green box in the image) with a Gaussian function to extract the FWHM. Scale bar: 10 mm (red).

Given the excellent photoluminescence properties of (GABA)_2_PbBr_4_, its potential application in X‐ray imaging was further evaluated. A flexible composite film of (GABA)_2_PbBr_4_ embedded in PDMS (denoted as (GABA)_2_PbBr_4_@PDMS) was fabricated, exhibiting good uniformity and optical transparency under visible light illumination, with a thickness of ≈0.6 mm (**Figure** **5**a). In addition, the composite demonstrated excellent mechanical flexibility, as shown in Figure S6 (Supporting Information). Under UV excitation, the film emitted bright white fluorescence (Figure 5b). Benefiting from the encapsulating protection of the organic PDMS matrix, the composite film maintained stable and bright emission even in aqueous environments. Figure 5c shows the radioluminescence (RL) intensity of the flexible film under various X‐ray dose rates. As the dose rate increased, the RL intensity steadily enhanced, demonstrating good response characteristics. Further analysis revealed a clear linear correlation between the RL intensity and X‐ray dose rate (Figure 5d). Moreover, upon termination of X‐ray exposure, the RL signal rapidly decayed to 0.58% of its initial intensity within just 40 ms (Figure 5e), indicating the material's fast response and low afterglow—favorable for high‐speed imaging applications.

Finally, an X‐ray imaging system based on the (GABA)_2_PbBr_4_@PDMS film was constructed to evaluate its spatial resolution. As illustrated in Figure 5f, the film emits fluorescence upon X‐ray irradiation of an object, which is then captured by a camera. X‐ray imaging of a standard line pair phantom (Figure 5g; Figure S7, Supporting Information) revealed a spatial resolution exceeding 10 LP mm^−1^. In addition, the (GABA)_2_PbBr_4_@PDMS film enabled clear visualization of fine structural details, such as the spring inside the fountain pen, the microchip, and the internal circuitry of the remote control, as illustrated in Figure 5h, with well‐defined object outlines and high image contrast. As shown in Figure 5i, pixel intensity analysis of the microchip image was used to fit the point spread function (PSF) of the intensity profile, yielding a spatial resolution better than 175 µm. In summary, the (GABA)_2_PbBr_4_@PDMS flexible composite film not only exhibits excellent scintillation characteristics—high sensitivity, low afterglow, and fast response—but also demonstrates great potential for use in flexible, high‐resolution X‐ray imaging applications.

In summary, large, high‐quality 2D organic–inorganic hybrid perovskite single crystals of (GABA)_2_PbBr_4_ were successfully synthesized via an in situ seed‐assisted cooling method. The unique structural integration of hydrogen‐rich organic GABA cations with heavy lead halide layers endows the material with excellent dual‐mode radiation detection performance. X‐ray diffraction confirms the highly oriented layered structure of the crystals, with a narrow half peak width of 12.81″ along the (004) plane, indicating superior crystallinity and structural order.

Optical characterization reveals a wide bandgap of ≈3.0 eV and strong excitonic emission, accompanied by pronounced self‐trapped exciton behavior. Transient photoluminescence and temperature‐dependent studies further elucidate the exciton dynamics, demonstrating the material's intrinsic photophysical advantages. These features result in an impressive γ‐ray response with an energy resolution of 9.0% at 662 keV and a high light yield of up to 10 695 ph MeV^−1^.

Importantly, the material exhibits excellent n/γ PSD performance, with a maximum FOM of 1.74, enabling complete separation of fast neutron and gamma ray signals. This characteristic addresses a long‐standing challenge in radiation detection: organic scintillators excel in PSD but lack gamma energy resolution, while inorganic scintillators offer high gamma sensitivity but poor neutron discrimination. The (GABA)_2_PbBr_4_ hybrid effectively bridges this gap through its dual‐functional design.

Furthermore, flexible composite scintillation films based on (GABA)_2_PbBr_4_ demonstrate high spatial resolution in X‐ray imaging, reaching up to 10 LP mm^−1^, highlighting the material's multifunctionality beyond neutron and gamma ray detection. Overall, (GABA)_2_PbBr_4_ emerges as a versatile and powerful candidate in the emerging field of advanced scintillation materials.

## 3 Experimental Section

### Materials

Lead(II) bromide (PbBr_2_, 99%), hydrobromic acid (HBr, 48%), γ‐aminobutyric acid (GABA) and paraxylene (≥99.0%)were purchased from Aladdin Chemistry Co., Ltd. Acetone was obtained from Sinopharm Chemical Reagent Co., Ltd. PDMS prepolymer and the curing agent were supplied as two‐part liquid component kits from Dow Corning (Sylgard 184). All reagents were used as received without further purification.

### Synthesis of (GABA)_2_PbBr_4_ Single Crystals

High‐quality (GABA)_2_PbBr_4_ single crystals were synthesized via an in situ seed‐assisted cooling crystallization. Specifically, γ‐aminobutyric acid (30 mmol, 3.0936 g) and lead(II) bromide (15 mmol, 5.5052 g) were dissolved in 50 mL of 48% HBr aqueous solution in a 50 mL Erlenmeyer flask. The mixture was stirred at 60 °C overnight to obtain a saturated precursor solution. After filtration, the clear solution was held at 60 °C for 2 h, followed by rapid cooling to 30 °C at a rate of 5.0 °C h^−1^ to induce the precipitation of microcrystals. The temperature was then raised to 58 °C at a rate of 0.5 °C h^−1^ and maintained for 6 h to dissolve excess crystals, leaving behind 1–2 seed crystals ≈1 mm in size. Subsequently, the solution was rapidly cooled to 56 °C to form a supersaturated solution containing seed crystals. Crystal growth was carried out by slowly cooling the solution from 56 °C to room temperature at a rate of 0.025 °C h^−1^. The resulting single crystals were collected, washed with warm acetone, and vacuum‐dried at 50 °C for 24 h.

### Fabrication of (GABA)_2_PbBr_4_@PDMS Flexible Film

The flexible composite film was fabricated via a drop‐casting technique. The PDMS matrix was prepared by mixing the two components of Sylgard 184 in a 10:1 weight ratio, supplemented with a small amount of *para*‐xylene as a dispersant. Ball‐milled (GABA)_2_PbBr_4_ powder was subsequently incorporated into the matrix under vigorous stirring to achieve a mass loading of 25%. After stirring for 6 h to ensure homogeneous dispersion, the mixture was rapidly injected into a mold and cured at 60 °C for 12 h to obtain the final flexible film.

### Structural and Optical Characterization

Single‐crystal XRD was conducted using a Cu Kα radiation source equipped with a liquid nitrogen cooling system. Powder and single‐crystal XRD patterns were collected using a BRUKER D8 Advance diffractometer operated at 40 kV and 40 mA with Cu Kα_1_ radiation (λ = 1.5406 Å). The scanning range was set from 5° to 50°, with a scanning rate of 5° min^−1^. Rocking curve measurements were also performed on the BRUKER D8 Advance system. UV–vis–NIR absorption and transmission spectra were recorded using a PerkinElmer Lambda 1050+ spectrophotometer, and optical bandgaps were estimated via Tauc plots. Steady‐state and power‐dependent PL spectra were measured using an Edinburgh FLS1000 fluorescence spectrometer. TRPL and temperature‐dependent PL spectra were obtained using an Edinburgh FLS980 steady‐state/transient fluorescence spectrometer.

### Radioluminescence and Scintillation Measurements

RL spectra were collected under X‐ray excitation from an Amptek Mini‐X2 tube (50 kV, 80 µA), with spectral acquisition via a TRIAX spectrometer. Pulse height spectra were recorded at room temperature under ^137^Cs, ^22^Na, ^241^Am, and ^57^Co γ‐ray sources using a Hamamatsu CR173 PMT powered by an ORTEC‐556 high‐voltage supply. Crystals were wrapped with Teflon tape (except for the face coupled to the PMT) and optically coupled using silicone oil. The (GABA)_2_PbBr_4_ single crystal used for γ‐ray and neutron detection measurements has dimensions of 28 × 7 × 3 mm^3^. For γ‐ray and neutron detection measurements, a single crystal with dimensions of 28 × 7 × 3 mm^3^ was employed. The absolute light yield was determined using the relative pulse height method with a commercial NaI:Tl scintillator as the standard reference. Pulse height spectra for both the (GABA)_2_PbBr_4_ sample and the NaI:Tl reference were recorded under ^137^Cs irradiation (662 keV) with identical settings. The full‐energy peak channel numbers (*Ch_sample_* and *Ch_ref_*) were extracted from Gaussian fits of the spectra. The absolute light yield (*LY_sample_*) was calculated using the following equation:

(8) $$LY_{sample}=LY_{ref}\times \frac{Ch_{sample}}{Ch_{ref}}$$

where *LY_ref_* is the light yield of NaI:Tl (38 000 ph MeV^−1^).

### Pulse Shape Discrimination

PSD measurements for neutron and γ‐ray signals were performed using a Hamamatsu CR173 PMT and a CAEN DT5720 digitizer with a sampling rate of 250 MS s^−1^.

### X‐ray Imaging

The X‐ray dose rate was calibrated using a commercial calorimeter. Imaging experiments were conducted using a digital camera to capture scintillation emission from the composite film under X‐ray irradiation.

## Conflict of Interest

The authors declare no conflict of interest.

## Supporting information



Supporting Information

## Data Availability

The data that support the findings of this study are presented in the article and Supporting Information. Additional data related to this study are available from the corresponding author upon request.
