# Supplementary material for: Structure‐Function Integration in 2D Hybrid Perovskite for Fast Neutron and Gamma Ray Discrimination
Source: Adv Sci (Weinh). 2025 Dec 14;13(13):e18905. doi: 10.1002/advs.202518905 (PMC12955889; doi:10.1002/advs.202518905)
Supplement: Supplementary file 1 — Supporting Information [file ADVS-13-e18905-s001.docx]

**Supporting Information of**

**Structure-Function Integration in 2D Hybrid Perovskite for** **Fast Neutron and Gamma Ray Discrimination**

Yingming Wang^1,2^, Lingyan Xu^1,2^*, Chongqi Liu^1,2^, Lu Liang^1,2^, Binghui Zhang^1,3^, Zhentao Qin^1,2^, Lixiang Lian^1,2^, Wei Zheng^1,2^, Yanyan Lei^1,2^, Qinzeng Hu^1,2^, Shuai Song^1,2^, Chaopeng Mi^1,2^, Tao Wang^1,2^, Yadong Xu^1,2^, Gangqiang Zha^4^, Wanqi Jie^1,2^

^1^State Key Laboratory of Solidification Processing, School of Materials Science and Engineering, Northwestern Polytechnical University, Xi’an 710072, China

^2^MIIT Key Laboratory of Radiation Detection Materials and Devices, School of Materials Science and Engineering, Northwestern Polytechnical University, Xi’an 710072, China

^3^Science and Technology on Thermostructural Composite Materials Laboratory, School of Materials Science and Engineering, Northwestern Polytechnical University, Xi’an 710072, China

^4^Research and Development Institute of Northwestern Polytechnical University in Shenzhen, Shenzhen 518057, China

*Corresponding author: xulingyan@nwpu.edu.cn

**Table of Contents**

[Fig. S1. Optical microscopy showing the layered texture along the (004) direction. 1](#_Toc215436394)

[Fig. S2. Significantly distorted octahedral framework with a Br-Pb-Br bonding angle of 146.0°. 2](#_Toc215436395)

[Fig. S3. X-ray radioluminescence(RL) spectra of (GABA)_2_PbBr_4_ single crystals. 3](#_Toc215436396)

[Fig. S4. Pulsed height spectrum of NaI single crystals at 662 keV γ-rays from ^137^Cs source. 4](#_Toc215436397)

[Fig. S5. Pulse height spectra of (GABA)_2_PbBr_4_ single crystals at 59.5 keV γ-rays from the ^241^Am source (a) and 122.1 keV γ-rays from the ^57^Co (b) source. 5](#_Toc215436398)

[Table S1. Comparison of scintillation properties of (GABA)2PbBr4 with other representative commercial and reported scintillators. 6](#_Toc215436399)

[Fig. S6. Mechanical flexibility of (GABA)2PbBr4@PDMS composites. 8](#_Toc215436400)

[Fig. S7. Physical image of a standard wire pair card in visible light. 9](#_Toc215436401)


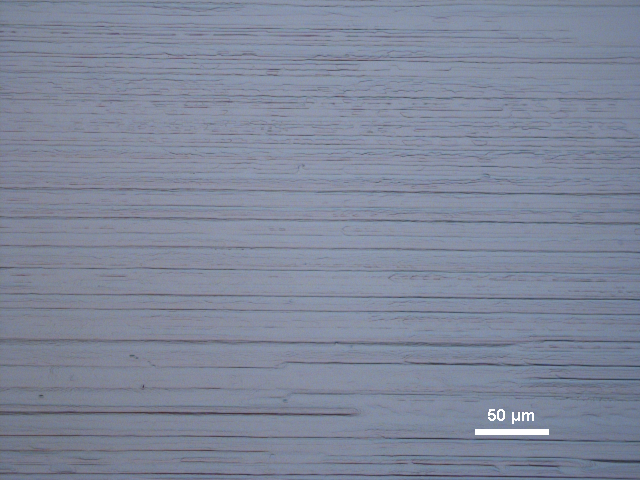


Fig. S1. Optical microscopy showing the layered texture along the (004) direction.


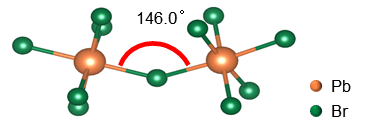


Fig. S2. Significantly distorted octahedral framework with a Br-Pb-Br bonding angle of 146.0°.





Fig. S3. X-ray radioluminescence(RL) spectra of (GABA)_2_PbBr_4_ single crystals.


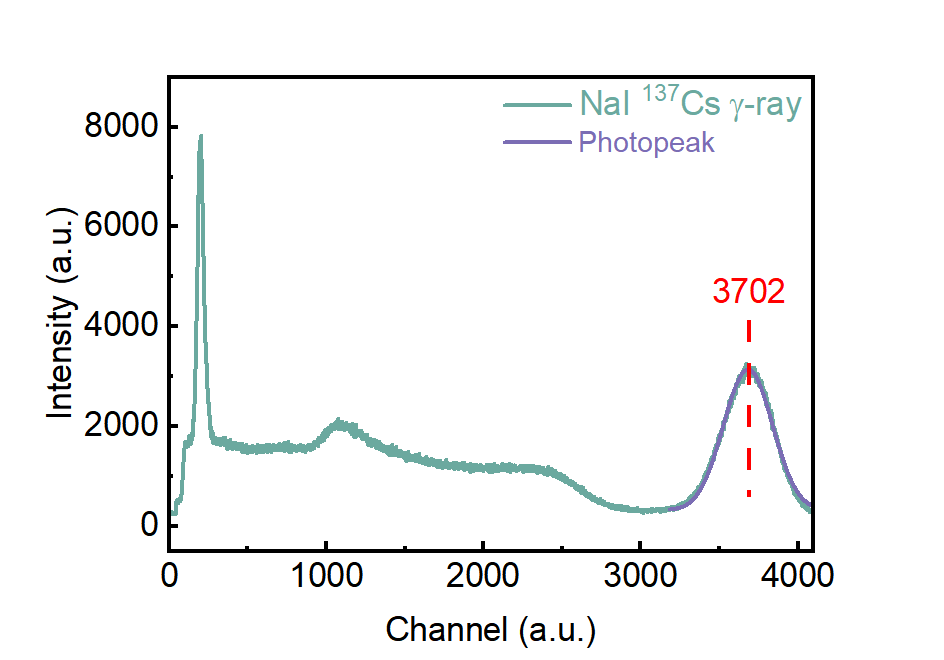


Fig. S4. Pulsed height spectrum of NaI single crystals at 662 keV γ-rays from ^137^Cs source.


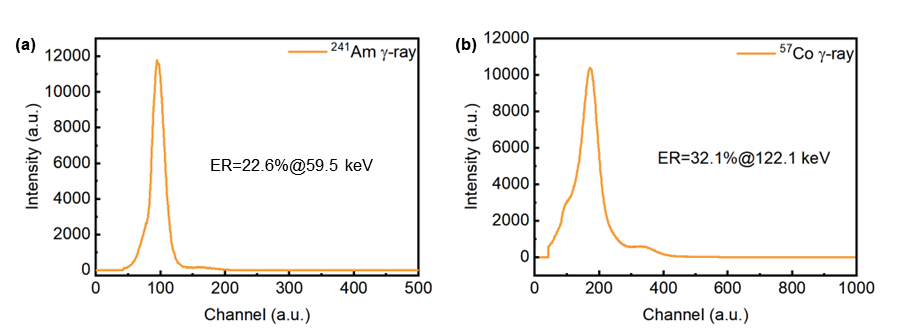


Fig. S5. Pulse height spectra of (GABA)_2_PbBr_4_ single crystals at 59.5 keV γ-rays from the ^241^Am source (a) and 122.1 keV γ-rays from the ^57^Co (b) source.

Table S1. Comparison of scintillation properties of (GABA)2PbBr4 with other representative commercial and reported scintillators.

| Material types | Materials | Light yield (ph MeV^-1^) | Energy resolution(%) | FOM | Neutron types | References |
| --- | --- | --- | --- | --- | --- | --- |
| Organic | EJ-276 | 8,600 | - | 2.0 | Fast | [1] |
|  | EJ-301 | 12,000 | - | 2.5 | Fast | [2] |
|  | BC-537 | 9,200 | - | 1.4 | Fast | [3] |
|  | Stilbene | 14,000 | - | 3.5 | Fast | [4] |
|  | Anthracene | 20,000 | - | 1.53 | Fast | [5] |
| Inorganic | NaI：Tl | 38,000 | 6.5 | - | - | [6] |
|  | Cs_2_LiYCl_6_ | 20,000 | 3.6 | 3.33 | Thermal | [7] |
|  | Cs_2_LiLaCl_6_ | 35,000 | 3.4 | 1.48 | Thermal | [8] |
|  | K_2_LiCeCl_6_ | 21,000 | 16 | 0.81 | Thermal | [9] |
|  | Cs_2_LiLaBr_6_ | 60,000 | 2.9 | 1.9 | Thermal | [10] |
|  | Cs_2_LiYBr_6_ | 24,000 | 4.1 | 1.23 | Thermal | [11] |
| Organic/inorganic hybrid perovskite | PEA_2_PbI_4_ | 1,000 | 35 | - | - | [12] |
|  | BM_2_PbBr_4_ | 3,190 | 19.5 | - | - | [13] |
|  | (BZA)_2_PbBr_4_ | 3,700 | 8 | - | - | [14] |
|  | BA_2_PbBr_4_ | 10,150 | - | - | - | [15] |
|  | Li-PEA_2_PbBr_4_ | 19580 | 9.5 | - | - | [16] |
|  | Li-BA_2_PbBr_4_ | 8800 | 10.9 | - | - | [16] |
|  | PEA_2_PbBr_4_ | 14,000 | 29 | - | - | [17] |
|  | PEA_2_PbBr_4_ | 31,300 | 14.3 | 0.73 | Fast | [18] |
|  | PEA_2_PbBr_4_: Zn | 41,000 | 4.84 | 0.85 | Fast | [18] |
|  | PEA_2_PbBr_4_: Sb | 40,400 | 5.65 | 1.03 | Fast | [18] |
|  | PEA_2_PbBr_4_:Eu | 30,000 | 15.57 | 0.66 | Fast | [18] |
|  | PEA_2_PbBr_4_:Cd | 28,500 | 18.6 | 0.74 | Fast | [18] |
|  | PEA_2_PbBr_4_ | 20,500 | - | 0.86 | Fast | [19] |
|  | (GABA)_2_PbBr_4_ | 10,695 | 9.0 | 1.74 | Fast | This work |


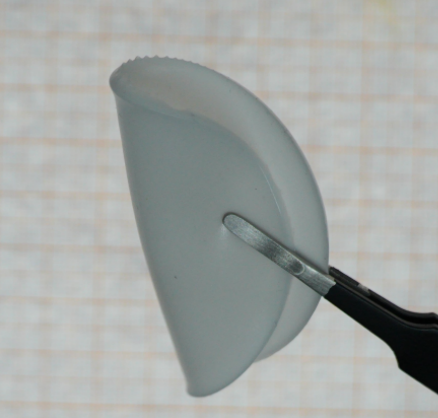


Fig. S6. Mechanical flexibility of (GABA)2PbBr4@PDMS composites.


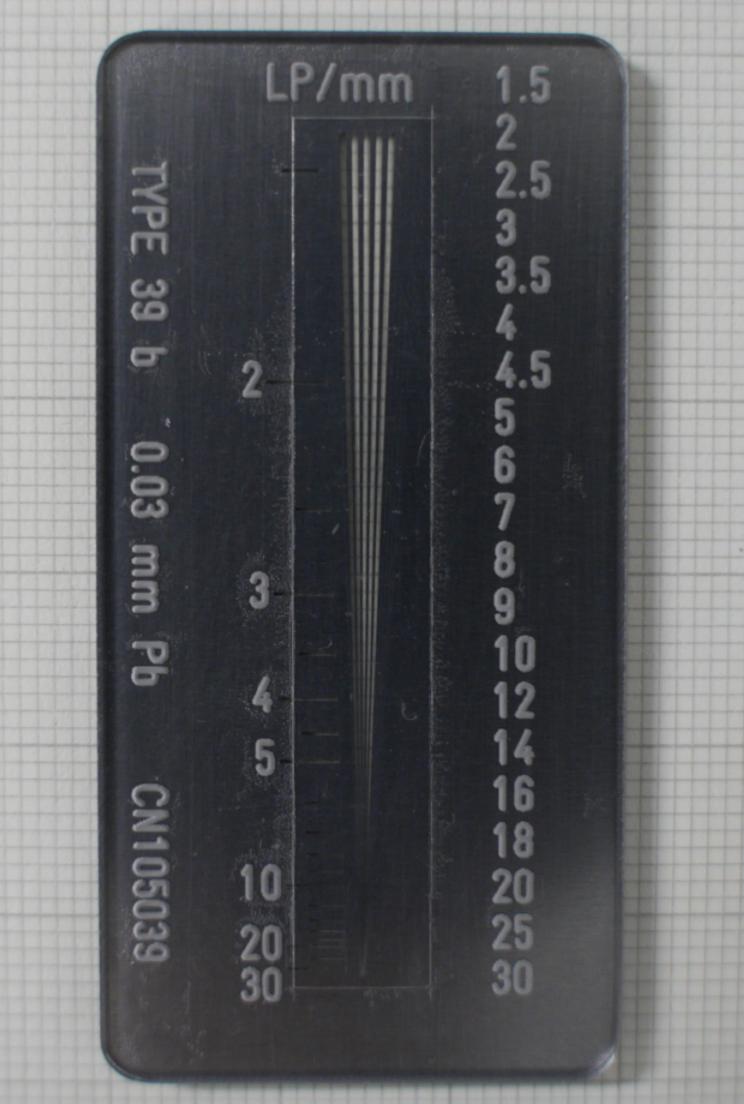


Fig. S7. Physical image of a standard wire pair card in visible light.
